# Trehalose Effect on The Aggregation of Model Proteins into Amyloid Fibrils

**DOI:** 10.3390/life10050060

**Published:** 2020-05-13

**Authors:** Eleonora Mari, Caterina Ricci, Silvia Pieraccini, Francesco Spinozzi, Paolo Mariani, Maria Grazia Ortore

**Affiliations:** 1 Department of Life and Environmental Sciences, Polytechnic University of Marche, I-60121 Ancona, Italy; emari1607@gmail.com (E.M.); c.ricci@univpm.it (C.R.); f.spinozzi@univpm.it (F.S.); p.mariani@univpm.it (P.M.); 2 Department of Chemistry “Giacomo Ciamician”, University of Bologna, I-40126 Bologna, Italy; silvia.pieraccini@unibo.it

**Keywords:** small angle X-ray scattering, circular dichroism, spectroscopy, amyloid, trehalose, protein solvation, lysozyme, insulin

## Abstract

Protein aggregation into amyloid fibrils is a phenomenon that attracts attention from a wide and composite part of the scientific community. Indeed, the presence of mature fibrils is associated with several neurodegenerative diseases, and in addition these supramolecular aggregates are considered promising self-assembling nanomaterials. In this framework, investigation on the effect of cosolutes on protein propensity to aggregate into fibrils is receiving growing interest, and new insights on this aspect might represent valuable steps towards comprehension of highly complex biological processes. In this work we studied the influence exerted by the osmolyte trehalose on fibrillation of two model proteins, that is, lysozyme and insulin, investigated during concomitant variation of the solution ionic strength due to NaCl. In order to monitor both secondary structures and the overall tridimensional conformations, we have performed UV spectroscopy measurements with Congo Red, Circular Dichroism, and synchrotron Small Angle X-ray Scattering. For both proteins we describe the effect of trehalose in changing the fibrillation pattern and, as main result, we observe that ionic strength in solution is a key factor in determining trehalose efficiency in slowing down or blocking protein fibrillation. Ionic strength reveals to be a competitive element with respect to trehalose, being able to counteract its inhibiting effects toward amyloidogenesis. Reported data highlight the importance of combining studies carried out on cosolutes with valuation of other physiological parameters that may affect the aggregation process. Also, the obtained experimental results allow to hypothesize a plausible mechanism adopted by the osmolyte to preserve protein surface and prevent protein fibrillation.

## 1. Introduction

A polypeptide chain in the folding process can achieve ordered or disordered structure, depending on the amino acid sequence, and on the physical and chemical features of the environment in which the protein is expressed. In the last decades misfolding of proteins and their aggregation in amyloid fibrils has attracted much attention, in particular from research areas studying neurodegenerative diseases. Amyloid fibrils are in fact commonly recognized as a result of protein misfolding and have been observed both in vitro and in vivo, where their accumulation and deposition into insoluble plaques and intracellular inclusions are the hallmark of several diseases known as amyloidosis. Alzheimer’s, Parkinson’s, and Huntington’s diseases are some of the approximately fifty amyloid diseases described to date. Amyloid fibrils, grown from proteins having different size, stability, and charge, share a common structure composed by repetitive sheets in which monomers are joined by hydrogen bonds across β-strands.

In non-pathological conditions, numerous stabilizing forces contribute to maintaining the correct folding of the protein. On this issue, hydrophobic interactions play a very important role: non-polar amino acids tend to minimize exposure to a polar solvent, such as water, thus locating themselves within the protein structure, while polar and charged amino acids are exposed to the surface of the protein, establishing close interactions with the solvent [1]. In stressful situations, such as extreme variations in temperature and pH, increased ionic strength or mechanical agitation, every protein can lose its native conformation, experiencing the possibility of rearranging into a different conformation or aggregating with other proteins in solution [2,3,4,5]. For example, in native conditions a protein has a limited degree of movement and flexibility that can be remarkably increased by increasing the temperature up to the conformation in which the protein exposes on its surface non-polar amino acids. Hence, the protein is allowed to establish protein-protein rather than protein-solvent interactions, with consequent modifications in aggregation and precipitation [6]. Also the presence of salts can mediate protein pathway of aggregation through ion interactions (Debye-Hückel screening or electroselectivity series), or by influencing water-solute interactions (Hofmeister series) [7]. These effects are then a combination of electrostatic and solute-solvent interactions, although many other exogenous and endogenous factors related to the specific protein and environment give their contribution [8,9,10].

In response to environmental stress, a variety of small molecules, classified as osmolytes, accumulate in cells. These natural species, including amino acids, sugars, polyols, but also tertiary sulphonium and quaternary ammonium compounds, present different interactions in the cellular context. While stabilizing osmolytes have been observed to be preferentially excluded from the protein surface [11,12], denaturing osmolytes like urea exhibit preferential binding to both native and disordered states of the protein [13,14]. However, recent Molecular Dynamics results suggest that the differences between protein shell and bulk compositions, in terms of water and cosolvents/cosolutes concentration, exhibit relevant changes with solvent composition, temperature, and protein nature [15]. These results prompt that the role played by a cosolute can change according to its absolute concentration in the environment, as well as according to temperature and to other chemical physical parameters, and it is exactly what osmolytes can do in vivo [16]. Given the osmolytes ability of maintaining the correct folding of proteins, these compounds have been extensively studied in recent years for their application as therapeutic agents in neurodegenerative diseases [17,18,19]. In particular, trehalose, among others, is able to preserve protein structural integrity, reduces aggregation of pathologically misfolded proteins, and improves the clearance of the mutant proteins which act as autophagy substrates when aberrant protein deposition occurs [20].

Several advances are made to understand and get detailed insight into the structural basis and mechanism of amyloid fibrils formation, cytotoxicity and therapeutic approaches to combat them [21,22,23,24,25,26,27,28]. In particular, when considering results collected on trehalose in the context of protein fibrillation, attention has to be paid also to the external environment characterized by a defined ionic strength and subjected to a particular redistribution of water molecules in proximity of protein surfaces. In particular, physiological ionic strength, while somewhat variable, averages around 155 mM, and for this reason it is important to investigate similar experimental conditions.

In this work, the competitive effect of trehalose and ionic strength has been investigated in relation to fibrillation of two model proteins: hen egg white lysozyme and bovine pancreatic insulin.

Specific protocols have been exploited to ensure the conversion of the proteins from their soluble forms into highly ordered, beta-sheeted, fibrillar aggregates under various conditions. The two proteins have been selected for their availability and for their different chemical characteristics.

Lysozyme, a glycosidase of 129 amino acids, in its native conformation has a globular shape. Its isoelectric point is at about 11.2, at physiological pH it is positively charged, and it shows a α+β conformation, with approximately 35% of the amino acids involved in α-helical structures and 10% involved in β-sheet structures, depending on the environment and buffer conditions. The structural stability is achieved, among the different forces involved, by four disulphide bridges [29,30]. Lysozyme fibrillation in presence of chaotropic anions of the Hofmeister series changes, and the timing of the lag phase is considerably reduced [31,32] even for relatively low concentrations of salt in solution (<300 mM) [33].

Insulin is a small peptide hormone produced in the β cells of the pancreatic islets of Langerhans. It is made up of two peptide chains, composed of 21 amino acids and 30 amino acids each. The two chains are linked by two disulphide bridges, while a third disulphide bridge resides inside a single chain. Insulin native form is mainly constituted by α-helical structures, and its isoelectric point is at about 5.4, leading its charge at physiological pH to be negative. It has been reported that its fibrillation is favored by NaCl that induces subtle structural changes pushing aside the N- and C-terminal segment, leading to the exposure of highly hydrophobic inner core [34]. The structural tweaking of insulin allows to recruit monomeric units for initial inter-molecular hydrophobic clumping that in turn leads to rapid association of insulin into amyloid fibrils. The propensity of insulin to fibrillate, can cause, besides pathological, also biomedical and biotechnological complications of high relevance, considering its wide therapeutic use in diabetes treatment and the requirement of functional insulin in each pharmaceutical dose [34].

In the light of renewed studies on trehalose behavior in water [35,36], or of trehalose preferential exclusion from protein hydration shell [37], we report on trehalose effect on amyloid fibrillation of two model proteins explored as a function of ionic strength. For this purpose, we have performed spectrophotometric experiments with Congo Red as specific amyloid dye, Circular Dichroism (CD), and Syncrothron Small Angle X-ray Scattering (SAXS) to monitor the temporal evolution of protein fibrillation, either in the absence or in the presence of NaCl.

## 2. Materials and Methods

### 2.1. Sample Preparation

Lysozyme from lyophilized white eggs was purchased from Sigma Aldrich, and it was used at a concentration of 3 mg/mL dissolved in glycine buffer (70 mM) at pH 2.3. Lysozyme solution was maintained at 4 ${}^{{}^{\circ}}$C overnight, and fibrillation was induced at 65 ${}^{{}^{\circ}}$C with gentle agitation according to literature protocols [33]. Bovine pancreatic insulin, purchased from Sigma Aldrich, was dissolved at a concentration of 3 mg/mL in HCl (25 mM) at pH 2.0, and kept at a temperature of 4 ${}^{{}^{\circ}}$C for one night. Subsequently, the fibrillation process was induced by bringing the sample to a temperature of 65 ${}^{{}^{\circ}}$C without agitating, and according to literature [38,39,40]. For both proteins, samples with or without trehalose and/or NaCl at various desidered concentrations were prepared.

### 2.2. UV-Visible Spectrophotometry

UV-Visible spectrophotometry, performed in presence of Congo Red (CR), was used to monitor the kinetics of aggregation of lysozyme and insulin into amyloid fibers, either in the absence or in the presence of trehalose and/or NaCl. Due to its high affinity for β-sheet conformations of fibrillated proteins [41], CR dye has been used as a diagnostic tool for amyloidogenesis since several decades. Indeed, tissues with amyloid aggregates assume, when stained with CR alkaline solutions, an intense red colour under sunlight. In addition, CR bonding with β-sheet structures induces a characteristic red-shifting of CR absorption maximum in Uv/Vis spectrum, from ≃490 nm to ≃540 nm. Although CR specificity in this field has been called into question [42], this approach might be considered still valid if used for semi-quantitative analysis and corroborated by other techniques.

Congo Red solution was prepared by CR dissolution in ethanol (80% *w*/*w*) and addition of NaCl up to saturation. The obtained solution was saturated with the dye powder and filtered with Millipore filters of pore size 0.2μm to remove possible aggregates.

Due to characteristic pH-dependent changes in the CR absorption spectrum, pH of lysozyme and insulin buffer solutions was brought at about 11.5 with a NaOH solution before UV/VIS analysis. Then, cuvettes for spectrophotometric measurements were prepared by adding 50 μL of the selected protein sample (pH ≃2), with 50 μL of the Congo Red solution, and 400 μL of the buffer used for each protein at pH 11.5. The amount of CR that we used for investigation was selected by performing several spectrophotometric measurements on different samples and comparing the obtained results. In particular, different volumes of Congo Red solutions were tested, that is, 10 μL, 30 μL, 50 μL, 70 μL, 90 μL, and we considered 50 μL as the most appropriate volume. After having verified the good reproducibility of the experiments, we carried out a semi-quantitative analysis finally providing the fraction of β structures formed in solution. Indeed, the ratio between absorption intensity at λ = 538 nm and at λ = 505 nm was calculated for each sample. These two wavelengths correspond to CR absorption maximum observed when it is associated with β structures, and to CR absorption peak when the dye is in its free state in solution. In order to compare this approach to the most commonly used protocol to quantify amyloid fibrils in solution, that is, Thioflavin T fluorescence measurements, the pattern of aggregation of the reference samples corresponding to lysozyme and insulin without cosolutes, was obtained by ThT measurements, too. The aggregation curves of the reference samples obtained by the two experimental methods were clearly overlapped (data reported in the Supplementary Materials).

For each result presented in this study, at least nine measurements were made, of which only the mean values are reported.

### 2.3. Circular Dichroism

CD measurements were performed at a room temperature of 20 ${}^{{}^{\circ}}$C with a Jasco J-715 spectropolarimeter, by using a circular quartz cell of 0.1 mm path-length. Protein concentration of samples was c=0.1mg/mL, and it was obtained by diluting, with the proper buffer, the protein solutions thermically treated as described in Section 2.1. Reported CD spectra were obtained by taking the average of three scans made at 50 nm/min from 260 to 190 nm. Each spectrum was corrected for the baseline by subtracting the spectral contribution of the buffer solutions, and spectral data are expressed in units of millidegree.

### 2.4. Small Angle X-ray Scattering

SAXS data were collected at the ID02 beamline at the European Synchrotron Radiation Facility in Grenoble, France, and at the Austrian beamline of Elettra Synchrotron in Trieste, Italy [43]. Measurements were carried out at 20 ${}^{{}^{\circ}}$C in capillaries of 1.5 mm outer diameter/0.01 mm wall thickness made from borosilicate (Hilgenberg, Maisfeld, Germany) enclosed within a thermostatic compartment connected to an external circulation bath and a thermal probe for temperature control. Two dimensional patterns were recorded by Pilatus3 1 M detector system based on the CMOS hybrid pixel technology. The data were stored in TIF format and then directly processed with FIT2D [44]. We measured each sample 20 times with an acquisition time of 20 s and a rest time of 40 s for each step after a negative control for radiation damage and checked for consistency with literature values before usage [45]. According to this procedure we aim to reduce the possibility to induce radiation damage. Raw data were radially averaged, and calibrated in absolute units (cm${}^{-1}$) by using a freshly prepared BSA solution (5.0mg/mL) in phosphate buffer and water. The sample-to-detector distance was set to 1.247 m, which provided wavenumbers *Q* by the equation Q=4πsinθ/λ, with 2θ being the scattering angle and λ equal to 1.54 Å the X-ray wavelength.

Both protein solutions (at concentration c=3.0 mg/mL) and buffers were measured at the same conditions concerning temperature and exposure time. The scattering curves have been normalized to the primary beam intensity, corrected for sample transmission and normalized to absolute scattering units. We carefully checked each set of scattering patterns and performed the average after a positive control over radiation damage. We did not observe radiation damage on samples presented in this study.

## 3. Results

### 3.1. LYSOZYME

#### 3.1.1. Spectroscopy Results

**Figure 1:**
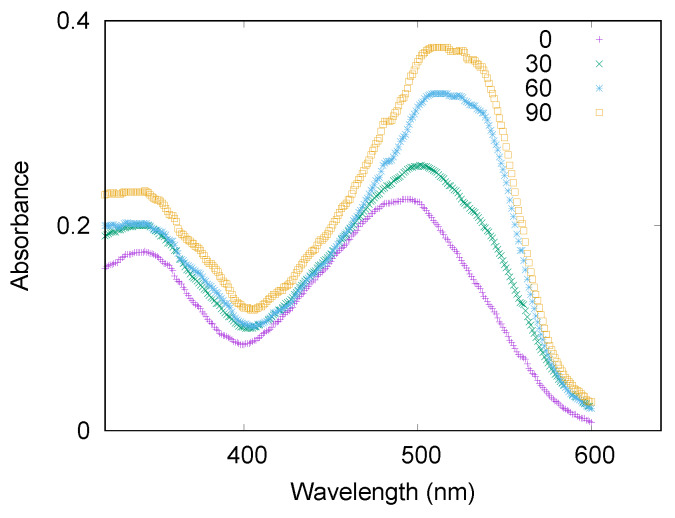
Absorption spectra of lysozyme at c=3.0 mg/mL, in the presence of CR and 50 mM NaCl. Spectra were recorded as a function of time, expressed in minutes in the legend, during amyloid aggregation induced by a temperature of 65 ${}^{{}^{\circ}}$C.

**Figure 2:**
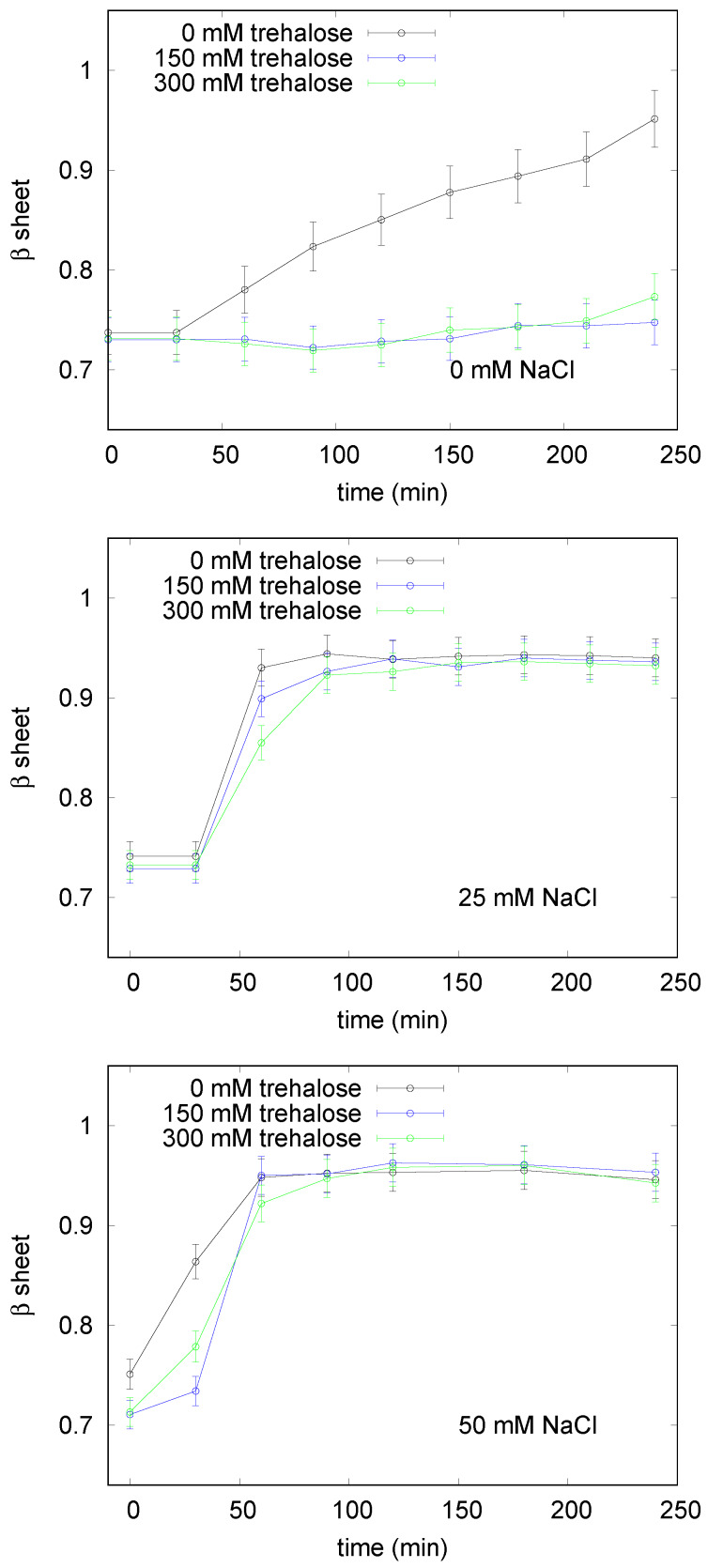
UV/VIS spectroscopy results on the effect of trehalose on lysozyme aggregation pattern. To monitor the relative amount of β-sheet structures in solution, the ratio between the intensity of the absorption peak due to Congo Red (CR) bound to fibrils and the one due to CR free in solution was calculated as a function of time. This ratio has been demonstrated to be proportional to β structures in solution (see Section 2.2 and [48]). Black circles indicate the sample without trehalose, blue squares the sample with 150 mM trehalose, and the green triangles the sample with 300 mM trehalose. Top: samples without NaCl, middle: with 25 mM NaCl and bottom: sample with 50 mM NaCl. The fibrillation kinetic of lysozyme in the absence of both trehalose and NaCl, top panel, reaches its plateau after 240 min, thus, for the sake of comparison, we report the first 240 min for all the investigated kinetics. The plateau values were confirmed, for each sample, after 24 h (data non reported). Error bars are estimated on the basis of the results of at least 9 replicas of the same experiment.

The influence of trehalose on lysozyme aggregation has been investigated analyzing the concomitant effectiveness of increasing ionic strength on the protein conformational evolution in time. The kinetics of the amyloid fibrillation for lysozyme in different selected conditions was at first monitored by UV/VIS absorption spectroscopy using CR as a probe for β-sheet formation. We underline that small aliquots of lysozyme in fibrillation conditions were kept from the stock solutions and then added with CR before UV/VIS analysis, thus we retain that the probe did not influence the fibrillation process. In Figure 1 few exemplifying absorption spectra are reported. It can be noticed that, as expected, an absorption peak due to free CR is present at ≃500 nm at time 0, that is, when no β structures except the ones of the native folded protein, are present in solution. Instead, as the aggregation process proceeds, this unique initial peak converts to a wider signal due to the simultaneous presence in solution of free CR and CR bound to β structures, these latter showing the typical absorption maximum at about 540 nm [34]. In the preparatory phase of the experiments, we successfully verified that the ratio between the area under the peak at higher wavelength and the area under the peak at lower wavelength, inside the error bars, was equal to the ratio between the corresponding intensities of the absorption peak due to CR bound to fibrils and the one due to CR free in solution. Thus, to estimate the relative amount of fibrils in solution, the ratio between the intensity of the absorption peak due to CR bound to fibrils and the intensity of the one due to CR free in solution was calculated. The obtained kinetic profiles describing lysozyme fibrillation are reported in Figure 2, where the relative amounts of β sheet structures are plotted as a function of time, in different panels for various salt contents. These results derive from at least 9 replicas of each experiment. Regardless trehalose content, lysozyme shows an aggregation rate that increases with NaCl content, especially in the first 60 min when the nucleation phase occurs together with the formation of the oligomers and/or of the protofibrils. This evidence suggests that counterions added to the solution, shielding the charges located on protein surfaces, can favor the initial aggregation, thus confirming that electrostatic factors are crucial for amiloidogenesis [46]. In particular, at pH 2.3 lysozyme charge corresponds to ≃18.8 elementary charges, hence the electrostatic repulsion between proteins is very high, and the addition of counterions in solution determines an immediate screening of the charges and a consequent decrease of their repulsion, favoring a fast nucleation. It can be noticed that the elongation phase presents a certain regularity in terms of timing in all the examined cases, indeed, with the exception of the sample without NaCl, the plateau occurs after about the first 90 min. Concerning trehalose effect, as shown by the upper panel of Figure 2, amyloid fibril formation results almost blocked by the osmolyte, either for a concentration of 300 or 150 mM. On the other hand, upon addition of NaCl 25 mM, trehalose inhibitory action drastically weakens. As shown in the central panel of Figure 2, and said before, a fast nucleation takes place in these conditions, and the osmolyte action results just in a slight slowdown of the elongation phase and a little reduction of final amount of fibrillated structures. At higher ionic strength (50 mM NaCl, bottom panel of Figure 2), the presence of trehalose as a cosolute slows down only the very beginning of the aggregation process (the first 45 min), and seems to be completely irrelevant for the final fibrillation. By observing the kinetics of fibrillation, it emerges that a strict direct proportionality between concentration of trehalose or NaCl, and effect on kinetic profile is lacking. Data show how trehalose, in the absence of NaCl, blocks the formation of amyloid fibers not strictly depending on its concentration, and its inhibiting action is greatly affected by the ionic strength regardless of salt concentration. On the other hand, the modest capability of the osmolyte to counteract the ionic strength effect is not always proportional to its concentration (see bottom panel in Figure 2). On the other side, in the presence of NaCl and regardless of its concentration, the inhibitory effect of trehalose appears to be affected. In fact, just a slight modification appears in the elongation phase, that is slown down at 25 mM NaCl with the higher amount of trehalose. We can resume that the amyloid aggregation of lysozyme is clearly influenced both by NaCl and by trehalose: protein fibrillation, observed by means of CR characteristic peak shift (Figure 2), is fastened by the presence of salt, even in low amount, and is blocked by the presence of trehalose, when the ionic strength is absent. Increasing the solution ionic strength, by adding NaCl, the effect of trehalose is invalidated and the kinetic pattern is similar to the one in absence of cosolutes, with just some traceable changes in the rate of the process.

**Figure 3:**
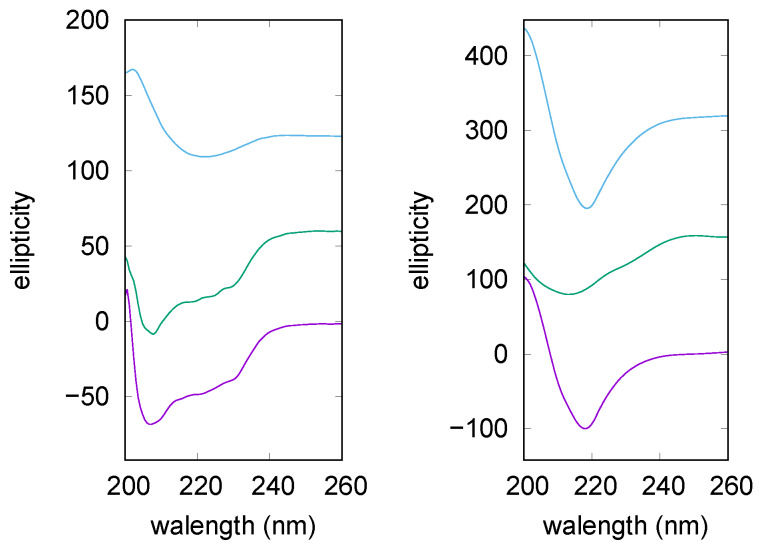
Circular Dichroism (CD) curves of lysozyme after 40 min (**left**) and after 120 min (**right**) at 65 ${}^{{}^{\circ}}$C. From bottom: lysozyme in solution without NaCl and without trehalose (violet), with 150 mM trehalose (middle, green) and 0 mM NaCl, with 150 mM trehalose and 25 mM NaCl (top, cyan). All the curves are scaled for clarity and smoothed approximating the data with a natural smoothing spline.

Trehalose ability to interfere with lysozyme aggregation, as well as its combined effect with NaCl, were validated by CD experiments, too. CD spectrum of native lysozyme (data not shown) is characterized by the presence of two minima at 208 nm and 222 nm [47], resulting by the combination of α and β components, with a prevalence of α-helical structure. The far-UV CD spectra measured on lysozyme solutions after different incubation times at 65 ${}^{{}^{\circ}}$C are reported in Figure 3. The two panels show CD spectra recorded in an intermediate phase (left panel) and in the final step (right panel) of lysozyme fibrillation, in the presence or the absence of trehalose and NaCl. It can be appreciated that after 40 min (left panel), both the pure lysozyme solution and the one added with trehalose (150 mM), present CD curves comparable to the one of the native protein, that is, lysozyme possesses in these conditions a secondary structure similar to the native one. On the contrary, in presence of trehalose plus NaCl (25 mM) a quite different band shape arises, indeed a negative CD band appears at around 215 nm as expected for β-sheet structures formation (see the top curve in the left panel of Figure 3) [39].

Thus, CD data confirm that, for lysozyme without trehalose and NaCl, amyloidogenesis is still in its lag-phase after 40 min of incubation, and the same occurs in presence of trehalose. Instead, the simultaneous presence of trehalose and NaCl results in accelerating the amiloidogenesis in accordance with UV data commented above. By observing the right panel of Figure 3, it can be seen that CD spectrum recorded on lysozyme after 120 min of incubation (bottom trace) shows significant changes. Indeed, the two negative bands centered at 208 and 222 nm, characteristic of the native state, convert into an intense negative single signal at ≃215 nm, indicating that the final fibrillation step is gained. A different trend is observed in presence of trehalose. In this case the CD curve (middle trace) retains a band shape comparable to the one of the native protein, thus confirming that the osmolyte can preserve lysozyme against self-aggregation. Addition of NaCl to the solution is able to nullify the trehalose effect, and a CD curve typically assigned to β-sheet structures is obtained again (see the top trace). Thus, CD data are in agreement with UV results previously described and collected on the basis of CR absorption, hence demonstrating that effects ascribed to trehalose or ionic strength are not related to the presence of the CR probe.

#### 3.1.2. SAXS Results

**Figure 4:**
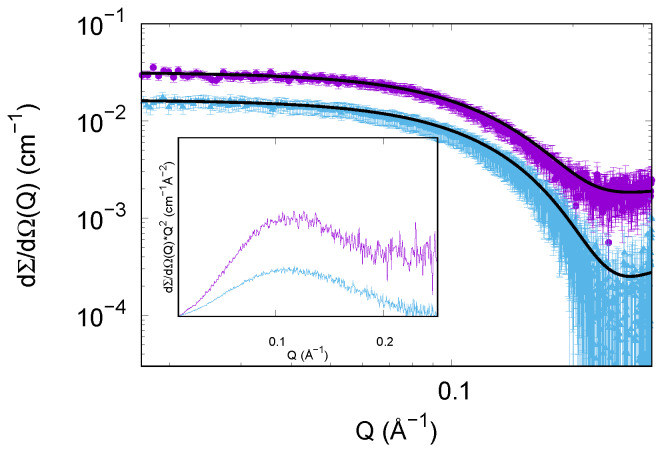
Small Angle X-ray Scattering (SAXS) curves of lysozyme at the beginning of the aggregation process at acidic pH: in absence (violet circles) and in presence (cyan triangles) of 150 mM trehalose. Fitting curves refer to 6LYZ PDB entry. In the inset, Kratky plots of the curves are shown in order to confirm protein compactness.

**Figure 5:**
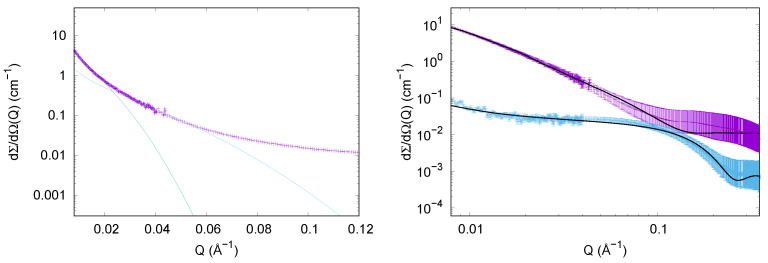
Experimental SAXS curves of lysozyme in different solutions at the end of the thermal treatment. Left panel reports SAXS experimental curve (violet points) and Guinier rod-like fitting of the final state of lysozyme aggregation, in absence of trehalose (cyan line corresponds to $R_{c}=30$ Å and green line corresponds to $R_{c}=77$ Å). Right panel reports SAXS experimental curves of lysozyme at the end of thermal treatment, in presence of trehalose (cyan points) and in absence of trehalose (violet points, curve shifted by a factor 2 for clarity). Continuous black lines represent the theoretical fitting obtained by considering disordered species in solution for lysozyme with trehalose, and by considering the simultaneous presence of cylinders and disordered species for lysozyme without trehalose.

In order to monitor also the three-dimensional structures resulting from lysozyme aggregation into amyloid fibrils, we performed synchrotron SAXS measurements at the beginning and at the end of the aggregation processes. SAXS data concerning native hen-egg-white lysozyme at pH 2.3 in glycine buffer with and without trehalose are reported in Figure 4. SAXS curve corresponding to lysozyme in presence of trehalose is comparable in shape to the one of the protein in the native state. In the inset SAXS data representation in the form of Kratky plot is presented to confirm the native conformation of the proteins. Kratky plots can qualitatively assess the flexibility and the degree of unfolding in samples: highly flexible proteins have a plateau in the Kratky plot at high *Q* values, while compact, globular proteins will show a bell-shaped peak [49]. The bell-shape of these Kratky plots clearly states that the proteins are in native conformation. Furthermore both the curves can be successfully fitted by using the form factor calculated from 6LYZ PDB entry, by taking advantage of GENFIT software [50], and considering a higher density hydration shell according to SASMOL approach [51]. The unique difference between lysozyme in solution with trehalose and without it resides in the different contrast of the protein with the medium, which is due to trehalose contribution to the bulk electron density. Monitoring the final stages of lysozyme aggregation (samples analyzed 3 h after the beginning of the amyloid aggregation process), the presence of aggregates has been verified, as it is evident in the left panel of Figure 5. A simple Guinier analysis [49] for globular objects in solution, can provide model-free information on the gyration radius $R_{g}$ according to the approximation $\frac{d\Sigma }{d\Omega }\left(Q\right)=\frac{d\Sigma }{d\Omega }\left(0\right)\;e^{-Q^{2}{R_{g}}^{2}/3}$. For native lysozyme, the gyration radius obtained is around 16 Å, corresponding to lysozyme monomer dimension. The same sample after the three hour temperature treatment, cannot be fitted by the classical Guinier approximation, but it has been successfully fitted by using the Guinier rod-like approximation ($\frac{d\Sigma }{d\Omega }\left(Q\right)=\frac{1}{Q}\;\frac{d\Sigma }{d\Omega }\left(0\right)\;e^{-Q^{2}{R_{c}}^{2}/2}$) that defines the cylindrical structure of the objects in solution (left panel of Figure 5). In particular, Guinier rod-like fitting accounted for the presence of at least two different families of elongated particles, the first one with a cross-sectional radius of about 77 Å, and the other one with a smaller radius of about 30 Å. This result confirms the amyloid aggregation of lysozyme, and the cross sectional radii are in agreement with those corresponding to mature amyloid fibrils (77 Å) and to protofibrils (30 Å) and obtained by atomic force microscopy (AFM) [52]. Although spectroscopy results already proved the increase of β structures as a consequence of lysozyme thermal treatment (see the black curve on the top panel of Figure 2), as well as ThT fluorescence spectroscopy experiments, SAXS results clearly demonstrate the three-dimensional features of the species in solution. SAXS data analysis proves the presence of at least two rod-like species that resemble fibrils and protofibrils and their simultaneous existence in solution resembles previous results on other amyloidogenic proteins [53,54]. Whilst Guinier rod-like approximation has the advantage to determine the average dimensions of elongated species without specific hypothesis, the whole SAXS curve is not fitted by this approximation.

Hence, we provided a further SAXS data analysis performed on all SAXS data points, and considering the simultaneous presence in solution of different species by GENFIT software [50]. This procedure has been already successfully applied to other cases of amyloidogenic proteins in solution [53], like α-synuclein [55] and amyloid β-peptide [23]. Results are reported in the right panel of Figure 5, and the theoretical curves correctly fit SAXS spectra, confirming the presence of different species in solution and providing their structural features. In the final investigated time step of lysozyme in solution without trehalose, according to SAXS data analysis two species are present: one has a cylindrical shape (whose radius is polydispersed around R=40 Å), confirming the fibrillar pattern, and the other is in a disordered conformation. In the final step of aggregation in presence of trehalose, instead the results are really different. In fact, the presence of trehalose inhibits the formation of fibrillar aggregates, allowing an analysis of SAXS data corresponding to the final state of the sample characterized uniquely by worm-like chains, fitted according to Pedersen Schurtenberger form factor model [56]. Two species of worm-like chains are present in solution: one with a low aggregation number (approximately 3), with a cross radius of 9.5 ± 0.5 Å, and another with a high aggregation number (approximately 10), with a cross radius of 15.8 ± 0.3 Å. It follows that trehalose prevents amyloid aggregation caused by the long thermal treatment. Due the large number of parameters needed to fit just one SAXS curve by two disordered species, parameters value do not provide a strong physical meaning, but merely confirm that trehalose prevents lysozyme fibrillation without preventing its unfolding due to both long term high temperature treatment and to very low pH.

### 3.2. INSULIN

#### 3.2.1. Spectroscopy Results

The kinetics of insulin denaturation and subsequent fibrillation are influenced by multiple parameters [38,40,57,58,59] and several cosolutes. In particular, it has been found that trehalose extends the lag phase of the fibrillation kinetic profile [58], thus slowing down amyloidogenesis. In the present work, we monitored insulin aggregation kinetic for further investigating trehalose effect also as a function of the ionic strength.

**Figure 6:**
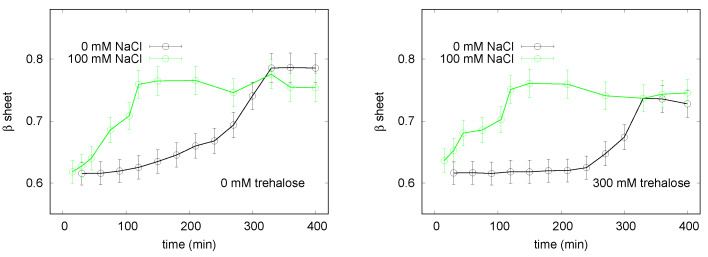
Analysis of the formation of β-sheet insulin structures in the absence and presence of trehalose (300 mM). The vertical axis reports an estimation of the amount of β-sheet structures in solution, obtained by the ratio between the intensity of the absorption peak due to CR bound to fibrils and the one due to CR free in solution (details are reported in Section 2.2). Black points indicate samples without NaCl, green circles represent the evolution of the sample with 100 mM NaCl.

Analogously to the experimental procedure used for studying lysozyme, different protein samples were prepared with or without trehalose and/or NaCl, and UV analysis was performed by using the CR dye as a probe for revealing β-sheet structures. The relative amounts of β structure plotted versus time and obtained for the investigated samples are shown in Figure 6. The curve related to insulin solution incubated without salt and osmolyte exhibits the characteristic lag phase, elongation phase and maturation phase corresponding to the final plateau. It can be noticed that, upon addition of trehalose, the lag phase is enhanced, and its duration varies from 80 min to about 240 min, as shown by the black traces reported in the two panels of Figure 6. In addition, a further effect concerns the final fibrillated structures, as the relative amount of detected β structures reduces of about 10% in the presence of the osmolyte. As expected, addition of NaCl to the insulin solution causes a significant acceleration of fibrillation, indeed a drastic shortening of the elongation phase is evidenced. As seen for lysozyme, also in the case of insulin the presence of salt counteracts the aggregation inhibiting trehalose properties. In fact, for insulin solutions containing NaCl the kinetics of aggregation observed in the presence or in the absence of trehalose are very similar, as shown by the green traces in the two panels of Figure 6.

**Figure 7:**
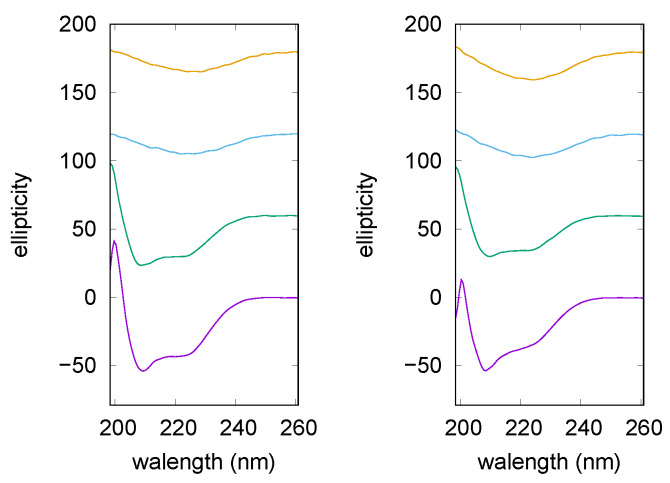
CD spectra recorded on insulin during incubation at 65 ${}^{{}^{\circ}}$C in the presence of NaCl 100 mM, without trehalose (left panel) and with 300 mM trehalose (right panel). The different curves correspond to different times: 0, 30, 60, and 90 min, from bottom to top.

To confirm the similar conformational evolution of these two latter considered cases, solutions were monitored during fibrillation by CD measurements. CD curves obtained at different times are reported in Figure 7. It can be seen that analogous CD band shapes emerge for the two examined samples, that is, for insulin with NaCl, in the presence (left panel) or in the absence (right panel) of trehalose. In both cases, after 30 min the native secondary structure is still detectable, while after 60 or 90 min the two characteristic minima are lost, and a single negative signal appears at ≃220 nm, in agreement with possible formation of β sheet structures.

CD data thus confirm that the presence of NaCl in solution erases the trehalose inhibiting effect on fibrillation.

#### 3.2.2. SAXS Results

**Figure 8:**
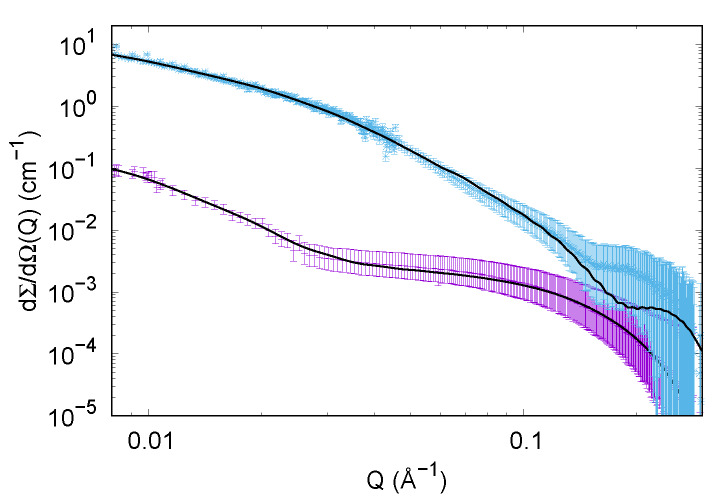
Experimental SAXS curves of insulin in different solutions at the end of the thermal treatment: in presence of trehalose (cyan points, curve shifted by a factor 4 for clarity) and in absence of trehalose (violet points). Continuous black lines represent the theoretical fitting obtained by considering disordered species and cylinders in solution, according to the details reported in Section 3.2.2.

The efficacy of trehalose alone on the aggregation pattern of insulin has been validated instead by SAXS. The insulin solutions exposed to thermal treatments, in presence and absence of trehalose, have been observed after 4 h of incubation, to check the final states. Figure 8 clearly shows how SAXS fingerprints of those final stages are different from each other. SAXS data corresponding to insulin without trehalose present a trend at low *Q* values that claims the presence of large aggregates. It should be noted that the same kinetics in presence of NaCl are not influenced by trehalose (data not showed). Hence a further SAXS data analysis was performed by GENFIT software [50] considering the simultaneous presence in solution of different species. Theoretical fittings together with experimental points are reported in Figure 8. SAXS data relative to insulin in solution without trehalose after the thermal treatment were fitted considering the predominant fibrillar state, populated by cylinders whose average radius measures ≃38 Å, and is highly polydispersed (about 30%), and a slight presence of disordered chains populated by less than 10% of the insulin monomers, whose cross section radius is about 20 Å. SAXS data corresponding to the sample subjected to the thermal treatment in presence of trehalose was successfully fitted by considering even in this case the same two different populations of objects in solution: polydispersed cylinders and disordered chains. However, we underline that in this case the predominant species is the disordered one (≃85%), while the cylindrical aggregates radii are wider in respect to the previous case (about 60 Å), probably suggesting a compaction among fibrils due to the presence of trehalose and consequent solvation changes.

## 4. Discussion and Conclusions

The process of formation of amyloid fibers is a complex phenomenon that involves numerous factors such as the intrinsic properties of the proteins considered and the chemical and physical conditions of the environment (for example, pH, temperature, ionic strength, concentration of the solution, agitation). In this study, the relationship between the fibrillation process of two model proteins, lysozyme and insulin, and the combined effects of trehalose and the ionic strength due to NaCl have been evaluated.

Trehalose, as an osmolyte, was known to increase protein stability and to move to higher values the melting temperature of proteins, thus favoring the native equilibrium of macromolecules [19]. Recent experimental data on the effect of trehalose on myoglobin thermal stability excluded the possibility that the protein can be stabilized by direct interaction with the trehalose molecules [60]. Instead, those results prompted that one to two molecular layers of water cover the protein surface and that the stabilizing trehalose molecules are generally located outside this hydration layer. According to this hypothesis, the trehalose-water matrix provides a stabilizing effect on the protein that increases with both the amount of trehalose-water around each protein (for a fixed water:trehalose ratio), as well as the concentration of trehalose in the surrounding solution [60]. The dynamics of the protein is thus, via the water layer, linked to the features of the trehalose-water matrix. These effects may allow the protein to be shielded from the environmental variations that induce denaturation, maintaining its own proper structure.

Our study confirms, for both the used proteins, trehalose effect against the fibrillation process, thus blocking, in the case of lysozyme, and inhibiting it, for insulin, as shown in Figure 2 and Figure 6. The different effect of trehalose for lysozyme and insulin may be ascribed to the different intrinsic structure, size, charge, and stability of the two proteins. Lysozyme molecular weight is about triple than insulin one, and lysozyme charge is 18.8 elementary charges, while insulin charge is 4.9 elementary charges at the investigated experimental conditions. Lysozyme, showing in its native state a large and compact hydrophobic core, has four disulfide bridges that increase its stability [61], while the two short insulin polypeptide chains, more exposed to solvent, are linked by one intra-chain and two inter-chain disulfide bonds [62]. The amyloid aggregation is subsequent to the weakening of protein intrinsic architectures, concomitant with a misfolding and/or unfolding of their native structures [63]. As a consequence, the mode and the speed of proteins fibrillation cannot but be correlated with their misfolding. Hence, although trehalose effect has been considered to be totally non specific and mainly due to the preferential hydration mechanism [64], a different efficacy against lysozyme and insulin fibrillation is evidenced, possibly correlated with their different intrinsic stabilities. Both lysozyme and insulin amyloid fibrils maintain the disulfide bridges of their native structures [62,65], hence disulfide bonds role is not easy to be inferred by our experiments.

The process of formation of amyloid fibers is influenced by the presence of NaCl in solution. Counterions screen protein surface charge, and as a consequence the coulombic repulsion between proteins decreases, favoring protein aggregation. The electrostatic contribution has been demonstrated to be an important factor in the amyloids formation [46], and an increase of salt concentration usually corresponds to a decrease of the lag time [33,34], as confirmed by our results (see Figure 2 and Figure 6), although an inverted behaviour has been recently observed on D76N mutant Beta2-microglobulin case [66]. These different effects of the addition of counter-ions in solution can be related to their different nominal concentration in solution, and to the influence of protein charge in the fibrillation process, which can vary according to the solution pH.

The main novelty of our study is in its focusing on the combined effect of trehalose and NaCl on protein fibrillation. In the case of insulin, the retarding effect of trehalose in fibrillation is completely canceled in the presence of 100 mM NaCl. In the case of lysozyme, the inhibitory effect of the osmolyte in fibrillation is greatly modified by salt, trehalose impact reduces uniquely to a detectable slowing down of the nucleation phase, that does not prevent at all the amyloid fibers formation. In addition, in this work the concomitant effects on lysozyme and on insulin fibrillation due to trehalose and NaCl have been followed also by SAXS. SAXS data analysis provides the amount of species in solution and their structural features, and reveals that when both NaCl and trehalose are in solution with proteins during the thermal treatment, huge amount of disordered species forms in solution. Hence, the effect of the thermal treatment combined with NaCl presence in solution, makes trehalose ineffective in maintaining the protein intrinsic structure and determines an aspecific aggregation of disordered proteins.

These experimental results invoke caution in all those attempts looking for chemical agents able to interfere with amyloidogenesis of proteins, expecially of proteins related to neurodegenerative diseases. The physiological ionic strength averages around 150 mM, and according to our results the promising effect of trehalose [19,20,59,67] fails in presence of NaCl even at concentrations lower than 100 mM. Other osmolytes have been found to positively interact against amyloidogenesis in vitro, as taurine, betaine, proline [39,68], but when in absence of a meaningful ionic strength in solution, those results should be considered with caution.

On the other hand, our results provide useful information to better understand the molecular mechanisms that trehalose adopts to act as a protective agent of biomolecules. The interaction of native proteins with the osmolyte endorse strengthening of hydrophobic interactions in the protein as a result of preferential exclusion of the osmolyte [35,39]. In absence of added salts, trehalose succeeds in maintaining the solvation sphere of the native protein thanks to the formation of a “water network”, allowing proper hydration. The presence of ionic strength reverses this process. Indeed, upon addition of salt, the water molecules are engaged in solvating the ions, in our case, Na${}^{+}$ and Cl${}^{-}$, located in particular in proximity of protein surface attracted by the electric charges. According to these considerations, the ions “steal” the water molecules that trehalose would like to organise onto the surface of the native protein, maintaining its hydration and, therefore, its correct folding.

The effect of NaCl on the system strengthens the “water entrapment” hypothesis for the osmolyte action, toward the “water replacement” one, that should not be affected by ions in solution.

Finally, trehalose, such as other carbohydrates, can accumulate in the cytoplasm of drought resistant cells, and is responsible for desiccation tolerance, as it is able to change the protein fibrillation pattern. Hence, exploitation of this osmolyte in several fields (food science, drug stability, bio-nanomaterials, etc.) could be very useful although a detailed description of its interaction with biomolecules is not yet completely established. The evaluation of trehalose ability to modify protein aggregation into amyloid fibrils in relation to the presence of NaCl is a new step toward the understanding of the interaction between the cosolute and the protein. Our study is an indirect confirm that the trapping of water molecules around the peptide can prevent structural damage [69] and consequent fibrillation, but noticeably underlines that this mechanism can be prevented by NaCl in solution. To conclude, we consider that in vitro experiments should take into account, step by step, nearly all the physiological factors that may possibly change their results when translated into an in vivo approach, and the ionic strength is a factor that cannot be neglected in protein fibrillation. 

## Supplementary Materials

The following are available at https://www.mdpi.com/2075-1729/10/5/60/s1, Figure S1: UV/VIS spectroscopy results obtained with Congo Red (CR) and Fluorescence spectroscopy results obtained with Thioflavin T (ThT) on lysozyme aggregation pattern. Figure S2: UV/VIS spectroscopy results obtained with Congo Red (CR) and Fluorescence spectroscopy results obtained with Thioflavin T (ThT) on insulin aggregation pattern.

## Abbreviations

The following abbreviations are used in this manuscript:

MDPI	Multidisciplinary Digital Publishing Institute
SAXS	Small Angle X-ray Scattering
CD	circular dichroism
