# Supplementary material for: Trehalose Effect on The Aggregation of Model Proteins into Amyloid Fibrils
Source: Life (Basel). 2020 May 13;10(5):60. doi: 10.3390/life10050060 (PMC7281244; doi:10.3390/life10050060)
Supplement: Supplementary file 1 [file life-10-00060-s001.pdf]

# SUPPLEMENTARY MATERIALS

## TREHALOSE EFFECT ON THE AGGREGATION OF MODEL PROTEINS INTO AMYLOID FIBRILS

Eleonora Mari[1], Caterina Ricci[1], Silvia Pieraccini[2], Francesco Spinozzi[1], Paolo Mariani[1] and Maria Grazia Ortore[1]

*1 Department of Life and Environmental Sciences, Polytechnic University of Marche, Ancona, Italy*

*2 Department of Chemistry "Giacomo Ciamician", University of Bologna, Bologna, Italy*

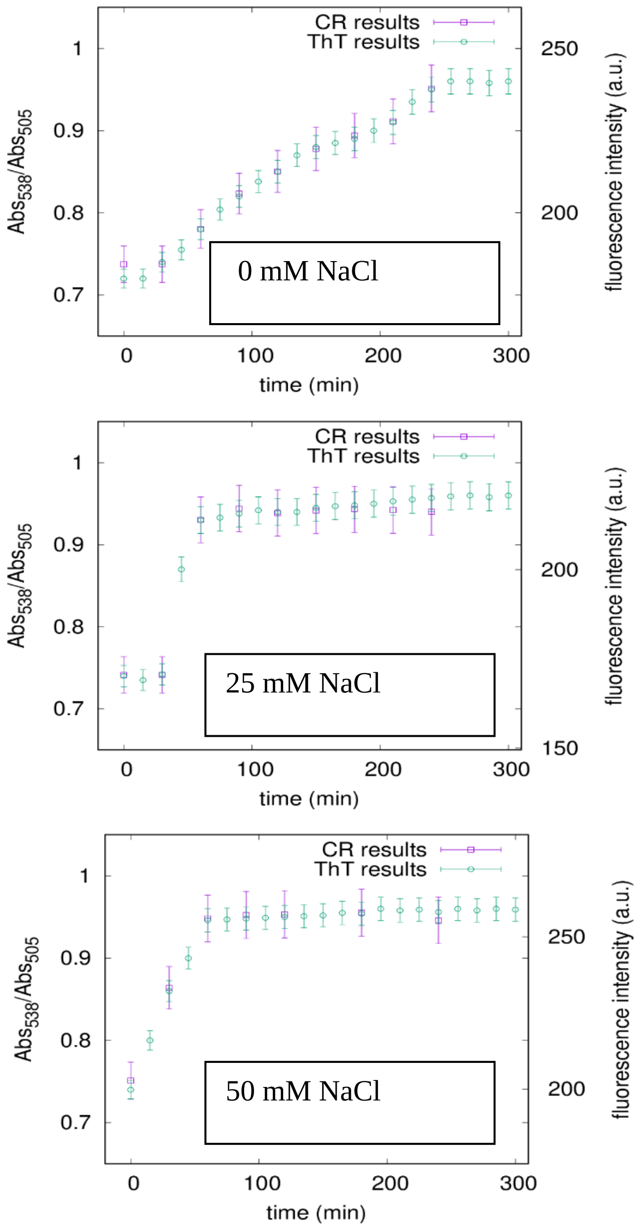


**Figure SM1.** UV/Vis spectroscopy results obtained with Congo Red (CR) and Fluorescence spectroscopy results obtained with Thioflavin T (ThT) on lysozyme aggregation pattern. From top to bottom, each panel refers to different NaCl content, as reported in the legend. CR results are those reported in Figure 2 of the manuscript, without trehalose. ThT results were obtained in order to test CR protocol efficiency. The fibrillation protocol of lysozyme was exactly the same for both protocols. Both CR and ThT have been added just before the measurements and were not pre-incubated with the samples. Error bars are estimated on the basis of the results of at least 3 replica.

Fluorescence measurements were performed with $\lambda_{ex}$ = 435 nm and $\lambda_{em}$ =485 nm in a standard right-angle geometry on a 0.5 × 1.0 cm PMMA semi-micro UV-cuvette (BRAND). During the measurements, the protein solution was not influenced by any mechanical stress (agitation or stirring).

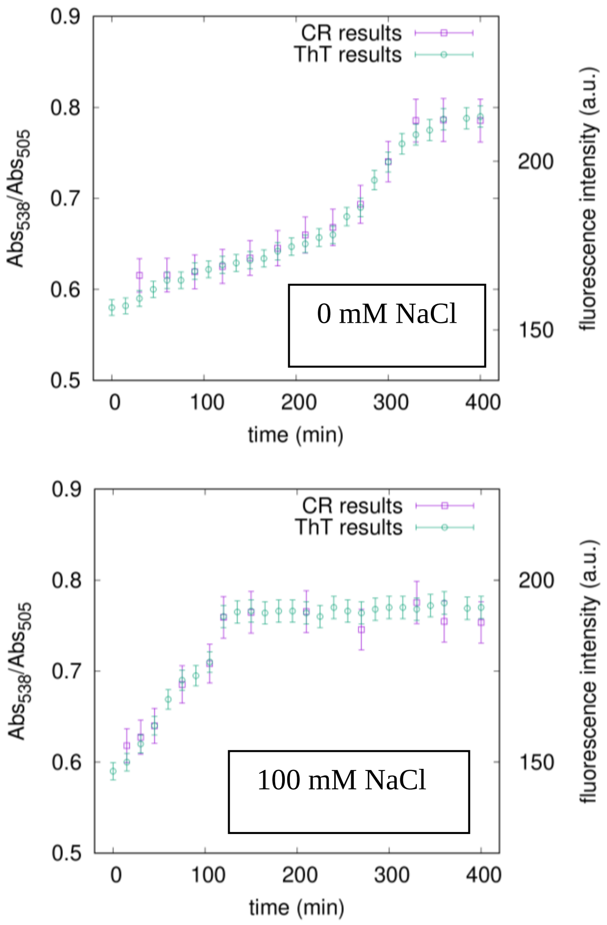


**Figure SM2.** UV/Vis spectroscopy results obtained with Congo Red (CR) and Fluorescence spectroscopy results obtained with Thioflavin T (ThT) on insulin aggregation pattern. From top to bottom, each panel refers to different NaCl content, as reported in the legend. CR results are those reported in Figure 6 of the manuscript, without trehalose. ThT results were obtained in order to test CR protocol efficiency. The fibrillation protocol of insulin was exactly the same for both protocols. Both CR and ThT have been added just before the measurements and were not pre-incubated with the samples. Error bars are estimated on the basis of the results of at least 3 replica. ThT fluorescence measurements were performed in the same conditions described in the caption of Figure SM1.
